# How the Pilot Low-Carbon City Policy Promotes Urban Green Innovation: Based on Temporal-Spatial Dual Perspectives

**DOI:** 10.3390/ijerph20010561

**Published:** 2022-12-29

**Authors:** Xianghua Yue, Shikuan Zhao, Xin Ding, Long Xin

**Affiliations:** 1 School of Economics and Management, Xiangnan University, Chenzhou 423000, China; 2 School of Public Policy and Administration, Chongqing University, Chongqing 400044, China; 3 School of Finance, Capital University of Economics and Business, Beijing 100070, China; 4 School of Economics and Management, Xinjiang University, Urumqi 830046, China

**Keywords:** pilot low-carbon city policy, green innovation, difference-in-difference, temporal-spatial heterogeneity, intermediary mechanism

## Abstract

Enhancing green innovation levels is an important objective of the pilot low-carbon city policy (PLCC) in China, but the spatial and temporal heterogeneity of the PLCC policy’s effect on green innovation is unclear. Based on panel data from 285 Chinese cities during 2005–2020, this paper assesses the impact of PLCC on regional green innovation using the difference-in-difference (DID) method. The empirical results demonstrate that the PLCC can obviously promote urban green innovation. In terms of the temporal dimension, the policy effect of PLCC on regional green innovation shows an inverted “U” shape and peaks in the seventh year after implementation. From the spatial dimension, the PLCC can promote surrounding cities’ green innovation through spatial spillover effects within 200 km, and the spillover effect decreases with increasing distance. Mechanism analysis indicates that the PLCC policy can promote regional green innovation by enhancing regional environmental regulations and alleviating financing constraints. This paper explores the temporal and spatial policy effects of PLCC, which can provide effective paths and policy recommendations for China to achieve its “dual carbon” goals.

## 1. Introduction

Recently, global climate change has caused a series of environmental problems such as global warming, extreme weather, glacier melting, and sea level rise, which seriously threaten the sustainable development of human society. Green innovation can achieve sustainable development by enhancing energy efficiency, reducing carbon emissions, and developing new technologies, and is increasingly becoming an important means to combat climate change. Meanwhile, to address climate change and achieve “dual carbon” goals, the Chinese government launched the Pilot Low Carbon Cities (PLCC) policy in 2010. The pilot cities have introduced low-carbon city development plans based on their industrial structure, resource endowment, and technological advantages, and most of these plans emphasize improving green innovation is an effective path to sustainable development of low-carbon cities. The PLCC policy means that we can achieve low carbon development in cities by improving energy efficiency, optimizing energy structure, eliminating high carbon industries, and adopting more environmentally friendly resources, and green innovation is an important driver to achieve this development model. The high priority given to the PLCC policy at the national level and the gradual expansion of the coverage of the pilot project demonstrate the importance of this pilot policy in promoting green development in China. The purpose of this paper is to explore the impact of the implementation of the PLCC policy on green innovation in Chinese cities.

The literature on environmental policy, green innovation, and PLCC is reviewed in this paper. The “Porter Hypothesis” typically serves as the foundation for literature on the effects of environmental regulation on technological innovation and productivity. For testing the weak “Porter Hypothesis”, scholars have discovered that more stringent environmental regulations force enterprises to spend more on R&D [1,2,3]. However, R&D spending is more an input to production processes, which do not immediately disclose innovation outputs, and R&D investment does not clarify the type of innovation as green innovation has a path-dependent influence. As a result, academics have begun to quantify local green innovation using more specialized and microscopic patent data [4,5,6]. The focus of studies on the potential influence of environmental policies on green innovation has likewise steadily migrated from the provincial to the urban level. Zhang et al. [7] investigated the impact of three environmental rules on green innovation and discovered that market-based and voluntary regulations are more effective in promoting green innovation than command-and-control environmental regulations. Based on data from patent applications filed by publicly traded firms, Cui et al. [8] evaluated the innovation impact of China’s pilot carbon emissions trading strategy and found that the policy promoted firm-level low-carbon technology innovation. To some extent, this reveals the micro-mechanics of city-level product switching in the face of environmental policies, i.e., cities may devote more resources to cleaner products while abandoning pollution-intensive productions. Tests of the strong “Porter hypothesis” are richer, but the findings are not consistent across the literature due to different measures of environmental regulation and differences in the samples selected. Some studies have found that environmental policy can promote productivity in specific industries [9,10]. Some academics have come to the contrary conclusion [11,12]. Other studies have also shown that environmental regulations might not always correlate directly with urban green productivity [13,14,15].

Research on low-carbon cities has been divided into two main directions. Some scholars believe that the implementation of PLCC policies can effectively promote urban pollution reduction and green development [16]. Based on data from 271 cities in China, Yan et al. [17] demonstrated that the implementation of PLCC policies effectively reduces haze pollution in China and that PLCC policies have spatial spillover effects. Cheng et al. [18] examined the effects of PLCC on green GDP at the city level using the difference-in-difference (DID) model and found that PLCC has an economy of scale effect and regional differences. The DID method was also used by Song et al. [19] to evaluate the impact of PLCC policy on urban ecological efficiency. He proposed that the PLCC could have significantly improved urban ecological efficiency and that the main intermediary mechanisms were technological innovation, industrial structure advancement, and energy utilization efficiency. This demonstrates that the PLCC’s implementation has some sort of promotional impact on urban green development. Another part of the study shows that the implementation of PLCC policy is not significant and even has the opposite effect. Fu et al. [20] believe that the PLCC policy’s influence on carbon reduction is not always immediate and that policy effects are more effective in the eastern region than the western region. Chen et al. [21] examined the impact of PLCC policy on urban carbon emissions from a spatial economics perspective and the empirical results showed that PLCC policies have a siphon effect and increase the carbon intensity of neighboring regions.

The above studies analyzed and discussed the PLCC policy from different perspectives; however, there are still limitations in the following aspects. First, although there is a rich literature to test the “Porter hypothesis,” early studies typically gauge a city’s investment in pollution control as a measure of environmental regulation intensity, and the more recent studies mainly examine a single environmental policy, rather than a comprehensive climate policy such as the PLCC. Second, although some literature has evaluated the effectiveness of pilot policies for PLCC using traditional policy evaluation methods, the selected assessment criteria have mostly focused on energy and carbon emission-related indicators, such as energy consumption, energy intensity, carbon emissions, and carbon intensity. Third, although the above indicators are direct manifestations of PLCC, existing studies have not explored how PLCC policy affects urban green innovation and have not captured their specific mechanisms from internal and external perspectives. Finally, despite the fact that previous research has mostly examined how PLCC regulations affect urban green innovation and how they work, few articles have been able to precisely portray the persistence of PLCC policy effects over time and their scope in space. The above research gaps will be the focus of this paper.

In response to the shortcomings of the prior literature, the key marginal contributions of this paper are as follows: (1) A useful addition to the literature on the implementation effect of this policy, this paper analyzes the policy effects of the PLCC from the perspective of urban green innovation. It offers a quantitative scientific basis for the evaluation of this comprehensive environmental policy. (2) After examining whether there is a policy effect of the PLCC program, the spatial and temporal heterogeneity of the policy effect is also examined, so that we can capture the duration and effective space of the policy effect more precisely. (3) We analyze and verify the impact mechanisms of the PLCC program on urban green innovation from two perspectives: environmental regulation intensity and alleviation of financing constraints.

The rest of the sections of this paper are organized as follows: The relevant literature is reviewed and research hypotheses are proposed in Section 2. Section 3 presents the construction of empirical models and data resources. Section 4 shows the empirical results and relevant analysis. Section 5 conducts the intermediary mechanism test. According to the analysis above, the research conclusions are drawn in Section 6, and some recommendations are also given based on these conclusions.

## 2. Theoretical Analysis and Research Hypotheses

### 2.1. The PLCC Policy and Green Innovation

China has proposed the PLCC program as a city-level environmental regulation strategy to carry out its climate action aims, which are characterized by weak constraints, sector specificity, and policy mix. Firstly, the PLCC policy is a loosely binding policy. The national government does not set deadlines for achieving PLCC goals; instead, this authority has been delegated to each pilot government. Additionally, each pilot city can support low-carbon work based on its specific circumstances, which is an exploratory attempt compared to other environmental regulation policies. Hence, whether the weak environmental policy can induce green technological innovation at the city level also remains to be tested. Secondly, the PLCC policy focuses on the low-carbon development of key energy-consuming and high-emission areas such as industry, buildings, transportation, energy supply, and waste management to achieve the city-level objectives of controlling carbon reduction and to induce green technological innovation in this process [22]. In contrast, the focus of other city-level pilot policies varies, but most do not target corporate green technology innovation. For example, the “Innovative City” pilot policy mainly promotes innovation in all aspects of economic, scientific, and technological, educational, and social development in cities through measures such as boosting investment in innovation resources and maximizing the distribution of innovation resources so as to improve the innovation environment [23,24]. The “Smart City” pilot policy focuses on the leap in urban governance mode brought about by information technology changes and achieves urban innovation in resource allocation, emerging industries, related technologies, and products through the application of smart devices and smart technologies to improve urban development patterns [25]. Finally, the PLCC policy is also characterized by a combination of policies. Based on the features of local economic growth, each pilot city has created a matching low-carbon city development program, technology, and industry advantages, which all encompass a variety of policy instruments, including market-based, command-and-control, and voluntary policies [26,27,28]. In addition, the pilot cities have implemented a number of green financial measures, including special funds, industrial subsidies, and credit preferences [29,30,31]. Thus, the policy is a combination of different policies to promote the decoupling of urbanization from carbon emissions.

By enhancing energy efficiency, lowering energy consumption, and cutting emissions during production, the PLCC policy will reduce both the city’s overall carbon intensity and total carbon emissions. The “Porter hypothesis” is realized as a result of this process, which is unavoidably followed by green production technology innovation. This will result in the creation of green technologies that address the demands of low-carbon development. According to the “Porter hypothesis”, there are two ways that environmental rules impact cities’ decisions about production. For starters, environmental legislation will have a cost-following effect, driving up the price of pollution control and emission reduction. Cities may tend to reduce R&D expenditure in the near term and move to other forms of investment as costs rise. For example, Luo et al. [32] show that firms will make different types of investments when faced with environmental policies, including increasing technology-based investments and financial-based investments. For another, well-designed environmental policies generate innovation compensation effects, which increase firms’ productivity and ultimately gain economic interest [33]. Under appropriate regulatory pressure, firms have an incentive to innovate technologically to increase energy efficiency and lower industrial process emissions while complying with mandated emission standards, ultimately achieving both environmental and economic benefits. Accordingly, we propose Hypothesis 1 as follows:

**Hypothesis** **1 (H1):** The implementation of PLCC policy can promote urban green innovation.

### 2.2. The Temporal Heterogeneity of PLCC Policy

The effect of PLCC on regional green innovation is highly heterogeneous at different temporal points after implementation. For one thing, the implementation of PLCC policy has a delayed policy effect. The impact of non-mandatory environmental regulation policies needs to go through a process of communication, implementation, and acceptance at all levels of government, and this process results in the policy effect not being immediately felt. From the available empirical evidence, Lu et al. [34] find that the positive effects of Chinese city pilot policies on investment, employment, output, productivity, and wages of new entrants are greater than those of existing firms. For another, the policy effects of PLCC will level off and disappear in the long run. In reality, in China’s progressive reform process, the early policy advantage of PLCC over other regions decreases over time. As “institutional rent” continues to evaporate, the impact of the PLCC policy on local green innovation will become increasingly heterogeneous over time. The French “Free Zone” policy for firms has been found to have a significant effect on firm location choice, and the policy has a positive effect on employment only in the short run [35,36]. Therefore, the actual effects of the same location-oriented policy can vary significantly from one point to another over time. We conclude that as the PLCC policy is promoted, its impact on local green innovation has an inverted “U”-shaped tendency, which rises first and falls subsequently.

**Hypothesis** **2 (H2):** The impact of PLCC policy on green innovation has a temporally heterogeneous effect.

### 2.3. The Spatial Heterogeneity of PLCC Policy

The PLCC policy can influence the green innovation level of neighboring regions through spatial effects. Firstly, the tournament competition among cities can give local government officials a strong incentive to emulate the low-carbon behavior of geographically and economically neighboring pilot cities. Specifically, China’s ecological civilization has been incorporated into local assessment indicators since 2012, and local governments are not only required to achieve GDP growth targets but also to meet environmental protection standards [37]. Second, the PLCC policy has boosted the overall capacity of urban green production and increased the cross-regional flow of green technological innovation components [38]. Meanwhile, the PLCC accelerates the process of imitation and cooperation in innovation activities among cities, making the flow of innovative R&D factors in green production-related fields more efficient [39].

In addition, it should be noted that the PLCC policy effect is spatially heterogeneous. Because spatial spillover effects are limited by distance [40], provinces with low-carbon city pilot policies benefit the most from positive spatial and economic growth driving effects. The PLCC policy is committed to jointly achieving carbon emission reduction and improving carbon efficiency, and the imitation effect of green technologies can realize their technology sharing over geographic and spatial distance. As a result, we argue that PLCC will have a greater impact on green innovation through spillover effects than through siphon effects [41]. In other words, the PLCC policy will have a positive green technology driving effect on nearby cities. When the maximum range of spillover impact is exceeded, the spatial effect of the PLCC also decreases. Therefore, the geographical influence on green technological innovation in the surrounding areas will be varied and exhibit a gradually declining trend as the distance to the PLCC grows. Based on the study above, we propose the following research hypothesis:

**Hypothesis** **3 (H3):** The impact of PLCC policy on green innovation has a spatially heterogeneous effect.

### 2.4. Mechanism Analysis of PLCC Policy on Green Innovation

The implementation of PLCC policy can influence urban green technology innovation through two mechanisms: external pressure and internal motivation. In terms of external pressure, the PLCC policy contains a package of environmental regulation policies [42]. With the implementation of various low-carbon environmental regulation policies, enterprises are forced to invest in green technology innovation passively due to environmental pressure. In terms of internal motivation, enterprises need green funding support for green technology development due to its risks and uncertainty [43,44,45]. The development of green finance has resulted in various forms of financial instruments, such as green loans and green bonds, which provide sufficient internal financial support for enterprises’ green R&D. Based on the preceding analysis, we choose environmental regulation and financing constraints as the proxy variables for the mediating mechanism.

The PLCC policies actually conform to the laws of the weak Porter hypothesis, that is, they encourage urban green innovation through environmental regulation tools. The different kinds of policy instruments used in PLCC are often categorized into three groups: directive tools, such as those used to reduce and eliminate outdated manufacturing capacity, green building energy efficiency, and automotive emission standards; market-based tools, mainly including policies such as carbon trading mechanisms, clean development mechanisms, and energy-saving pilots; and voluntary tools, such as pilot low-carbon transportation systems, low-carbon industrial parks, and zero-carbon buildings. Enterprises’ green innovation is impacted differently by several types of policy instruments. Comparing the three types of policies, the directive policy tools are the most severe, which could increase urban pollution control investment. Some studies have shown that urban pollution control investment can effectively induce invention patents with high innovation content [46,47] and have a strong contribution to the overall environmental efficiency in China [48]. The narrow “Porter hypothesis” suggests that flexible market-based instruments may be more conducive to inducing technological innovation due to their cost-effectiveness [49]. However, factors such as institutional architecture and the degree of marketization have an impact on how effective they are, and research confirms the role of market-based instruments in inducing innovation in Chinese firms [50,51,52]. Compared to the first two policies, voluntary-type policy instruments are the least constraining for firms and are still relatively little practiced in China, and their impact on firm innovation has been confirmed by several studies [53,54]. As a result, we propose this following hypothesis:

**Hypothesis** **4 (H4):** The PLCC policy can promote urban green innovation by strengthening environmental regulation.

Schumpeter’s innovation theory argues that the availability of finance is crucial to technological innovation. Due to return uncertainty, information asymmetry, and high regulatory costs, green innovation operations are vulnerable to significant external finance limitations, which will stifle enterprises’ innovation efforts [55]. Green technical innovation differs from typical technological innovation because it involves a significant initial capital investment, a lengthy profit cycle, and unpredictable risk. Therefore, in order to address issues with market failure that green innovation faces, such as environmental externalities, path dependency, and imperfect capital markets, specific financial support must be included. This suggests that more money must be invested for directional change innovation, and as a result, businesses may be more prone to have financial restrictions when developing green technology [56,57]. In reality, the PLCC has proposed various green finance policies, including tax advantages, special funds for low-carbon growth, sector-specific subsidies, and loans with advantageous interest rates. These green financial policies can direct more capital into green industries and environmental protection industries in order to highlight technological innovation breakthroughs. Accordingly, hypothesis 5 is proposed.

**Hypothesis** **5 (H5):** The PLCC policy can promote corporate green innovation by alleviating financing constraints.

**Figure 1:**
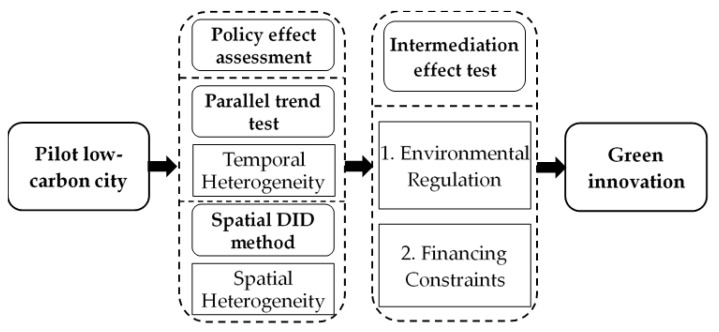
The PLCC policy effect on green innovation.

Based on the above analysis, the research framework of this paper is established, which is shown in Figure 1.

## 3. Model Construction and Variables Description

### 3.1. Model Construction

#### 3.1.1. Basic Model

The PLCC policy not only leads to regional differences between pilot and non-pilot cities, but also causes disparities in the pilot cities before and after the policy is implemented. As a result, the DID method is used in this article to evaluate how PLCC policy affects urban green innovation. The Chinese government announced three batches of PLCC policy in 2010, 2012, and 2017, which means that the traditional DID method is no longer applicable. Following the approach of Beck [58], this paper builds a progressive DID model to analyze how PLCC policy affects green innovation. The specific model is as follows:

(1) $$green_{it}=\beta_{0}+\beta_{1}policy_{it}+\rho X_{it}+\nu_{i}+\mu_{t}+\varepsilon_{ij}$$

In Equation (1), the $green_{it}$ stands for green innovation for city i in year t, and we use the number of urban green patent applications to represent the level of urban green innovation. The $policy_{it}$ represents the dummy variable for the PLCC policy, and indicates whether city i has implemented the PLCC policy in year t. $X_{it}$ denotes the set of control variables which affect green innovation in city i in year t; $\nu_{i}$ and $\mu_{t}$ represent urban fixed effects and time fixed effects, respectively; $\beta_{0}$ and $\varepsilon_{ij}$ represent the constant term and the random error term, respectively.

#### 3.1.2. Temporal Econometric Model

With reference to the event study approach (parallel trend testing) [59], the following econometric model is set up to test the temporal heterogeneity of the PLCC policy effects.

(2) $$green_{it}=\alpha_{0}+\prod_{k\geq -5}^{7}\alpha_{k}D_{it}^{k}+\lambda Z_{it}+\nu_{it}+\mu_{it}+\varepsilon_{it}$$

In Equation (2), $D_{it}^{k}$ represents the dummy variable for the implementation of the PLCC. It should be noted that due to the long sample period in this paper, we only examine the policy effects from 5 years prior to policy implementation to 7 years after policy implementation. If the time before the policy implementation is earlier than 5 years (−6, −7, −8, ……, −13 years), we merge these samples into 5th year before the policy implementation; if the time after the policy implementation is later than 7 years (8, 9, 10 years), we merge these samples into 7th year after the policy implementation. In this way, we assume that city i becomes a PLCC city in T, let k=t−T; when k≤−5, $D_{it}^{-5}=1$, otherwise $D_{it}^{-5}=0$. When k≥7, $D_{it}^{7}=1$, otherwise $D_{it}^{7}=0$. By contrasting the statistical and economic relevance of a parameter $\alpha_{k}$ in Equation (2), we can test the time variation of the policy effect of the PLCC.

#### 3.1.3. Spatial Analysis Model

The spatial DID model, unlike the conventional DID model, can manage any potential spatial association between variables while also decomposing the treatment effect into direct effect, spatial spillover effect (indirect effect), and total effect through parameter testing. Referring to the derivation process of Dubé [60] and Xin [61], a three-class spatial DID is constructed.

Firstly, a spatial lagged DID model is constructed as Equation (3).

(3) $$green_{it}=\rho Wgreen_{it}+\alpha_{1}policy_{it}+\alpha_{2}X_{it}+\mu_{i}+\varepsilon_{it}$$

Secondly, a spatial error DID model is constructed as Equation (4).

(4) $$green_{it}=\alpha_{1}policy_{it}+\alpha_{2}X_{it}+\mu_{i}+\mu_{it},\;\mu_{it}=\lambda W\mu_{it}+\varepsilon_{it}$$

Thirdly, spatial Durbin DID model is constructed as Equation (5).

(5) $$green_{it}=\rho Wgreen_{it}+\alpha_{1}policy_{it}+\beta_{1}Wpolicy_{it}+\alpha_{2}X_{it}+\beta_{2}WX_{it}+\mu_{i}+\varepsilon_{it}$$

In Equations (3)–(5), the variable symbols have the same mean as in Equation (1). W represents the spatial weight matrix, and two spatial weight matrices are chosen to express the spatial relationships of 285 cities in this paper. The one is the geographic distance spatial weight matrix *Wd*. We construct it using the inverse of the spherical distance between cities. The other is the economic distance weight matrix *We*. We take the difference between the mean GDP values of city i and city j from 2005 to 2020, and then using the inverse of the difference’s absolute value to get *We*. The spatial weight matrix *W* with the combination of different variables (Wpolicy, Wμ, and Wy) represent the impact of the spatial effect of that variable in the local city on the green innovation in the neighborhood.

The spatial DID model can assess the spatial effect of PLCC policy (including spatial spillover effects and spatial siphon effects), but it is difficult to capture the characteristics of the policy effects in terms of spatial distribution. To examine the regional heterogeneity of PLCC policy’s impacts on green innovation according to Li [62]’s research, we establish the following model:

(6) $$green_{it}=\beta_{0}+\beta_{1}did_{it}+\sum_{s=50}^{400}\delta_{s}N_{it}^{s}+\lambda Z_{it}+\nu_{i}+\mu_{t}+\varepsilon_{it}$$

Equation (6) shows a new set of control variables $N_{it}^{s}$, and s represents the geographic distance between cities (kilometers, s≥0), which is measured by the spherical distance between two cities. In particular, if a PLCC exists within a year’s spatial distance of a city i(s−50,s) in t year, so $N_{it}^{s}=1$, otherwise $N_{it}^{s}=0$. For example, $N_{it}^{50}$ indicates whether a PLCC exists within a spatial distance of no more than 50 km from city i in t year. Therefore, the coefficient $\delta_{s}$ of variable $N_{it}^{s}$ evaluates how the PLCC policy affects the neighboring cities’ urban green innovation after the implementation. In the specific regression analysis, this paper reports the regression results of Equation (6) when s=50,100,…,350,400, respectively, and tests the spatial heterogeneity of the PLCC policy effect by contrasting the statistical and economic relevance of a parameter $\delta_{s}$ at different thresholds.

### 3.2. Variable Description

**Figure 2:**
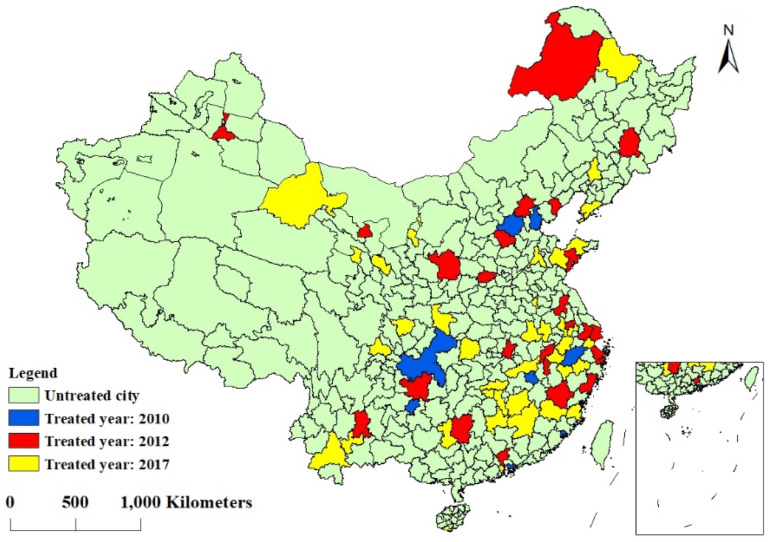
Spatial distribution of pilot low-carbon cities.

1. Independent variable (*green*). The level of patent technology is an important indicator of a region’s ability to innovate. This paper collects the number of green patent applications in Chinese cities to gauge urban green innovation.

2. Dependent variable (*policy*). The core explanatory variable is the dummy variable “whether or not a pilot low-carbon city was established in that year.” Since 2010, China’s National Development and Reform Commission has been actively promoting the PLCC program. The second and third batches of pilot member cities were added in 2012 and 2017, respectively. By the end of 2020, 87 Chinese cities will have joined the ranks of low-carbon city pilots. Specifically, since the implementation of the PLCC policy took place in July 2010, November 2012, and January 2017, considering that the first two batches of pilot policies generally took effect in the second half of the year and the lag of policy effects, we take 2011, 2013, and 2018 as the base period for policy implementation. In the empirical analysis, the years after being affected by the policy shock can be up to the 9th year, while the years before being affected by the policy can be up to 13 years ago. In addition, due to the quality and availability of data, 71 pilot cities were selected as the test group in this paper (Figure 2 and Table 1).

3. Control variables. To depict the PLCC policy’s impacting aspects on green innovation more precisely, the following variables are also controlled in this paper: (1) Economic development level (*pgdp*) The level of economic development will affect the regional science and technology innovation capacity. In order to determine the level of regional economic development, this article uses GDP per capita and its logarithm. (2) The level of R&D investment (*rd*). The proportion of each city’s R&D to urban GDP is used to calculate this indicator. (3) urbanization level (*urb*). The percentage of each city’s population that lives in an urban area is used to calculate this indicator. (4) Foreign Direct Investment (*fdi*). The amount of foreign direct investment in the GDP serves as a proxy for this control variable. (5) Government intervention (*gov*), measured by each city’s ratio of fiscal spending to GDP. (6) Human capital (*hum*). This control variable is measured by the ratio of teacher–students in universities. (7) The transportation infrastructure (*tra*) measured by the area of road coverage per capita in each region.

4. Statistical description. Taking the availability of the data into account, cities with significant data gaps are removed, and some missing data are processed using the interpolation approach. The final selection for the research sample includes 285 prefecture-level cities using panel data collected in China from 2005 to 2020. The empirical data are obtained from the China Research Data Service Platform (CNRDS) database, the China Science and Technology Statistical Yearbook, the China City Statistical Yearbook, and the China Statistical Yearbook. In addition, R&D investment, transportation infrastructure, and FDI data are collected from the National Bureau of Statistics, the statistical yearbooks of each prefecture-level city, and official websites. Meanwhile, the non-proportional data were logarithmized to remove the impact of the magnitude error prior to completing the empirical regressions, and the data involving the influence of price were treated at constant prices with 2005 as the base period. Table 2 displays the results of descriptive statistics for each variable.

## 4. Empirical Results and Analysis

### 4.1. Baseline Model Regression Results

Table 3 reports the regression results of Equation (1), where columns (1) and (3) do not control city and year fixed effects and columns (2) and (4) control city and year fixed effects. All results in Table 1 show that the coefficients of the dependent are positive at the 1% statistical level, which shows that PLCC adoption has a considerable beneficial impact on regional green innovation, and the results are still reliable when other control variables are included. Based on the results (4) in Table 1, the number of regional green patent applications increases by about 0.1613% when the PLCC starts to be implemented. Considering the continuity of the PLCC policy, this policy promotion effect is a rather significant increase for the development of green innovation in pilot cities. As a result, PLCC policy execution may considerably raise cities’ degrees of innovation in green technology, and hypothesis 1 is verified. Related studies have come to similar conclusions that PLCC policy can promote total factor productivity of firms, urban FDI, and thus urban green development [63,64]. The marginal contribution of this paper is to verify whether climate policy such as PLCC can support Porter’s hypothesis and promote urban green technology innovation.

### 4.2. The Temporal Analysis of PLCC Policy

**Figure 3:**
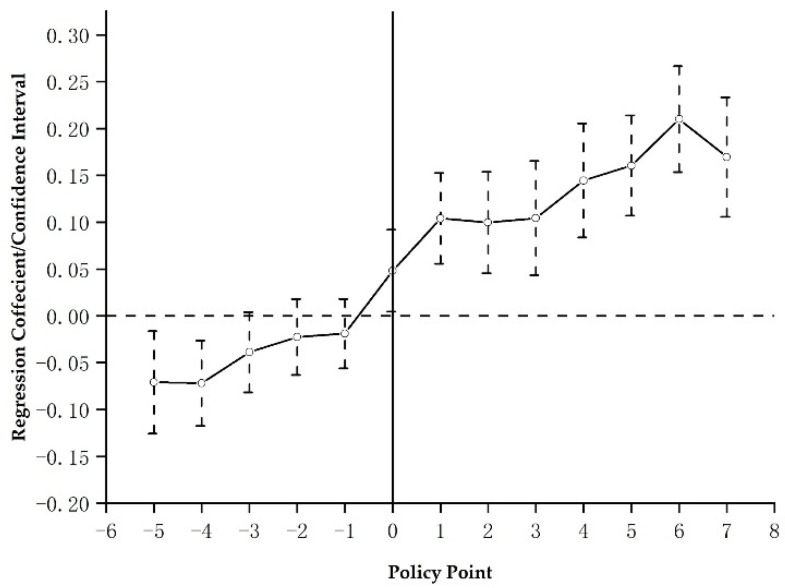
Temporal heterogeneity of the pilot low-carbon city policy effect.

To test the temporal heterogeneity of the PLCC policy effect on regional green innovation in hypothesis 2, we empirically test Equation (2). Figure 3 reports the regression coefficient $\alpha_{k}$ of variable $D^{k}$ in Equation (2) over time (with a confidence interval of 95%). It was discovered that the PLCC policy considerably encouraged green innovation in the host city from the first to the seventh years following its inception, and that the influence of the policy increased yearly. The PLCC policy’s boosting influence on green innovation does not become negligible in the host city until the eighth year. In general, with the implementation of the PLCC policy, the policy effect is characterized by an inverted “U” shape in the temporal dimension, which rises first and falls after the 6th year. Specifically, the promotion effect reaches its maximum in the 6th year after establishment and begins to fall after the 7th year after establishment, which indicates that there is a temporal heterogeneity of the PLCC policy effect. Hence, Hypothesis 2 is verified.

### 4.3. The Spatial Analysis of PLCC Policy

#### 4.3.1. Regression Results of the Spatial DID Model

Table 4 reports the regression results of the spatial models (Equations (3)–(5)) based on the geographic weight matrix *Wd* and the economic weight matrix *We*. The results in Table 4 show that the estimated coefficients (*policy*) of all two types of spatial matrices are significantly positive at the 1% level, which is consistent with the baseline regression results in Table 3. This indicates that the conclusion that the PLCC policy can promote urban green innovation still holds after controlling for the spatial correlation of the variables.

The spatial spillover effect exists in three aspects based on the spatially lag-term regression coefficients of each variable: First, for the spatially lagged term variable policy (columns (5) and (6)), according to the SDM-SDID model based on the Wd and We matrices, its coefficients are considerably positive at the 5% and 1% levels, respectively, suggesting that PLCC policy has a spatial effect. This means that cities with PLCC policies in place may not only advance green innovation locally but also foster its growth in cities that are close to each other economically and geographically. Second, based on *Wd* and *We* estimation, the coefficients of the spatially lagged error term (columns (3) and (4)) are both significantly positive at the 1% level in the SEM-SDID model, indicating that there is a spatial spillover effect of the random disturbance term factors and that the unobserved factors in cities with similar geographical or economic distance will have a positive impact on local green technology innovation. Finally, the coefficients in the SAR-SDID model and the SDM-SDID model based on *Wd* and *We* estimation are both significantly positive at the 1% level for the spatial lag variables of green innovation (columns (1) and (2)), indicating that there is a spatial spillover effect on the level of green technology innovation and that cities with higher levels of green technology innovation can drive the development of green technology in the nearby areas. In conclusion, green innovation has a significant geographical spillover impact that may be observed in both locations with equal geographic distances and those with stronger economic linkages.

#### 4.3.2. Decomposition of the Spatial Effect

The results of the spatial diagnostic test show that the optimal spatial econometric model is the spatial Durbin double difference model (SDM-SDID) based on double fixed effects. By using partial differentiation, the regression coefficients may be divided into direct impact, indirect effect, and total effect in order to more effectively capture the geographic effect characteristics of the PLCC policy. The regression results are shown in Table 5.

As shown in Table 5, we may derive the following conclusions from the decomposition outcomes of the SDM-SDID model. The PLCC policy has a major impact on stimulating green innovation in the pilot cities, as shown by the fact that all of its direct impacts are significantly favorable. Comparing the results of the benchmark regression coefficient, we find that the direct impact of PLCC policy in the spatial DID model is smaller, indicating that the baseline DID model estimation may overestimate the direct effect of PLCC policy due to ignoring the spatial dependence. Second, from the spatial spillover effect (indirect effect), the spatial spillover effect of green innovation driven by the PLCC implementation accounts for more than 50% of the total effect, which further affirms the spatial spillover effect of the PLCC policy implementation’s significant contribution to urban green innovation. Finally, the geographical spillover effect based on *Wd* is much bigger than the spatial spillover effect based on *We*, showing that the geographic distance is more favorable for the spatial spillover impact of PLCC cities, as seen by the variability of the spatial weight matrix. There are two possible reasons for these differences. Firstly, from the perspective of environmental regulation, regions with similar geographic distance tend to have similar levels of environmental pollution and face similar levels of environmental regulation accordingly. This results in a greater spatial demonstration effect of low-carbon city pilot policies among cities located close to one another. Second, in terms of knowledge spillover, green innovation, as a type of intellectual capital, is more likely to be transmitted between cities that are close geographically. In recent years, the level of ICT and transportation infrastructures has been greatly improved, and cross-regional R&D exchanges and technological cooperation are frequently carried out, which leads to the spatial spillover effect of green innovation between cities with closer geographical distance. In summary, the research hypothesis 3 of this paper can be verified.

#### 4.3.3. Spatial Heterogeneity Analysis

**Figure 4:**
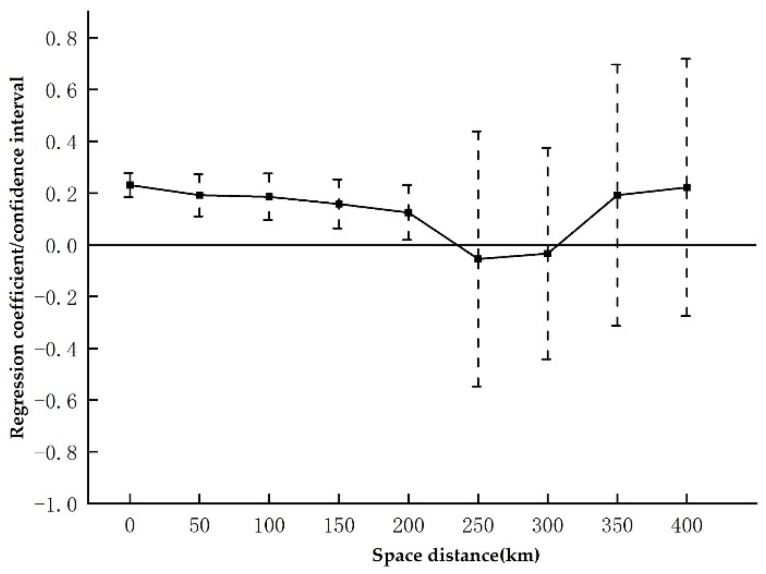
Spatial heterogeneity of low carbon city policy effects.

Based on the empirical findings in Table 4 and Table 5, we draw the conclusion that the PLCC policy has a sizable spatial spillover impact; nevertheless, a scoping study of the spatial spillover effect’s bounds is required. Figure 4 plots the trend of the coefficient of variation with spatial distance according to the estimation results of Equation (6) (with a 95% confidence interval). In particular, the promoting effect of the PLCC policy on green innovation in nearby cities shows a tendency to gradually diminish as the distance to the PLCC cities grows. Moreover, the distance to the PLCC will have a significant contribution to the green innovation of cities within 200 km; when the distance exceeds 200 km, the green innovation-promoting effect on the neighboring cities becomes insignificant again. This also verifies the spatial heterogeneity of the promoting effect of the PLCC policy on green technology progress in neighboring cities in Hypothesis 3.

This outcome is consistent with the expectations of the agglomeration economy theory. Due to their geographic and spatial proximity to the pilot cities, which are on the PLCC list, and the fact that these cities typically have high carbon emissions in terms of environmental pollution, the cities surrounding these pilot cities also absorb the transfer of high-pollution, high-energy, and high-emission industries from these cities. According to the viewpoint of economic agglomeration, low-carbon city pilot programs are frequently implemented in regional economic hubs. The growth of the central city also has a substantial spatial spillover effect, which stimulates the growth of nearby cities. The economic growth of the major city also has a substantial geographical spillover effect, resulting in the economic growth of other cities and giving such cities greater financial clout to carry out green technology R&D. In terms of technology spillover, cities closer to low-carbon pilot cities are more likely to accept technology transfer from the pilot cities and then learn, use, and re-invent green patents based on the local actual situation in order to improve their own green technology innovation level. In summary, the spatial spillover effect of PLCC policy decreases with increasing distance.

### 4.4. Robustness Tests

#### 4.4.1. Propensity Score Match (PSM) Method

PSM-DID is the strategy that is selected to reduce selection bias and the endogeneity problem that results in order to further regulate any potential variations between the PLCC and other cities (Table 6). First, to determine the propensity score for the entire sample, we first calculate the likelihood that each city will become the treatment group in turn using the logistic regression method. Then, using a suitable matching method, we identify the city with the closest propensity score as the treatment group’s control group. Finally, the matched control group and the treated group are used for DID estimation. The empirical results based on PSM-DID are basically consistent with Table 1. The conclusion that the PLCC policy can promote urban green innovation is verified again.

#### 4.4.2. Placebo Test

In this paper, two “counterfactual” methods were used to conduct placebo testing.

The treatment and control groups were firstly randomly assigned. The original treatment group’s PLCC-implementing cities were regarded as the new control group. We kept the time of PLCC implementation unchanged. If there were cities that implemented the PLCC policy in the year, then a new treatment group was randomly selected from the cities that had never established a PLCC policy in that year or before. In order to complete the placebo test, the column (4) in Table 1 was then re-estimated using the new sample. The above process was repeated 1000 times, and thus the estimated coefficients of the dummy variable policy could be estimated. The estimated results showed that the mean value of policy was −0.2915, which is much smaller than the 0.1613 estimated from the column (4) of Table 1. The results indicate that the policy effect of PLCC shows a significant locational orientation, and the policy effect is more pronounced in cities that have implemented PLCC policy.

The second method was to randomize the implementation time of the PLCC policy in advance. Assuming that the city that implemented the PLCC remains the same, if in reality the city implemented the PLCC policy in the year, then any year from the time range [2005, t−1] was randomly selected as the time when the city implemented the PLCC. Then, the coefficient of the variable policy can be re-estimated using the new sample in model (4) of Table 1. After repeating the above process 1000 times, the estimation results show that the mean value of the coefficient of the variable policy was 0.1112, which is about 31% lower than the estimation result of the column (4) in Table 1. Thus, randomly advancing the timing of the PLCC policy leads to a significant decrease in the policy innovation effect and the placebo tests also confirm that the establishment of PLCC policies does increase the green innovation of the host city from a counterfactual perspective.

#### 4.4.3. Transformation Sample and Outlier Test

The robustness tests were conducted in four aspects (Table 7). Firstly, we used the implementation year of the PLCC policy as the implementation time instead of delaying it by one year, and column (1) reports the corresponding test results. Second, by reducing the sample size, we accounted for the outliers in the number of green patent applications. We specifically eliminated the biggest and smallest 1% of all green patent applications, and then the indentation approach was added on top of that. The matching test results were then reported by column (2). Third, even when the acquired number of green patents was substituted for the independent variable in column (3), the regression result was still significant. Fourth, adjusting for additional fixed effects, province fixed effects, and year fixed effects were controlled by column (4), whereas column (5) concurrently controls province fixed effects, year fixed effects, province–years interaction fixed effects, and city fixed effects. Finally, we used the technique of substituting indicators for the explanatory variables by substituting the quantity of green patent acquisitions in cities for the quantity of green patent applications. The coefficients of the variable policy are still statistically significant, according to the regression findings of the aforementioned model.

## 5. Analysis of Intermediary Mechanisms

### 5.1. Intermediary Model Construction

The study presented above demonstrates that, to a certain extent, PLCC policy implementation can foster urban green innovation, and there are different dimensions of heterogeneity in the green innovation effects of this policy. As a city-based comprehensive environmental management tool, the PLCC policy adopts different types of policy instruments to promote local green technology development. On the one hand, as a comprehensive environmental regulation policy, the PLCC policy will strengthen environmental regulation, and the pollution control investment will increase correspondingly. On the other hand, it also strongly advocates supporting urban low-carbonization through financial instruments, which may also greatly alleviate enterprises’ financing constraints in green innovation. Then, financial instruments are also strongly advocated to support urban low-carbonization, which may also greatly alleviate the financing constraints of enterprises in green innovation and provide essential financial support for their green technology innovation. Therefore, this paper explores the effect mechanisms from two angles in order to further understand the processes by which the PLCC policy induces green technology innovation: the types of environmental regulation and the alleviation of financing constraints. Referring to Wang [65] for the test of the impact mechanism in the DID approach, this paper constructs the mediating effect model as follows.

(7) $$green_{it}=\alpha_{1}+\beta_{1}policy_{it}+\lambda X_{it}+\nu_{i}+\mu_{t}+\varepsilon_{it}$$

(8) $$M_{it}=\alpha_{2}+\beta_{2}policy+\lambda X_{it}+\nu_{i}+\mu_{t}+\varepsilon_{it}$$

(9) $$green_{it}=\alpha_{3}+\beta_{3}policy_{it}+\beta_{4}M_{it}+\lambda X_{it}+\nu_{i}+\mu_{t}+\varepsilon_{it}$$

where $M_{it}$ represents the mediating variable, which represents both environmental regulation (*reg*) and financing constraint (loan) variables. In this paper, the pollution control investment of each city is used to represent the intensity of environmental regulation. The year-end loan balance of financial institutions in each city is used to represent the intensity of financing constraints. All the original data are from the China City Statistical Yearbook. In the empirical test, if $\beta_{1},\beta_{2}$, and $\beta_{4}$ in Equations (7)–(9) are significantly positive, and the estimated coefficient value of *policy* becomes smaller or less significant in Equation (9), it indicates the existence of a mediating effect.

### 5.2. Intermediary Mechanism Analysis

Table 8 shows the empirical results of the mediation mechanism test. After adding the mediating variables into the empirical model, the regression coefficients in columns (3) and (5) are 0.1112 and 0.1255 at the 1% significance level, respectively, which are significantly smaller than the regression coefficient of policy in column (1) (0.1613). The regression coefficients of policy in columns (2) and (4) are also significantly positive, which implies that a large part of the policy effect of PLCC is realized through pollution control investment and green loans. Above all, the PLCC policy can promote green innovation by strengthening urban environmental regulation and alleviating financing constraints, and research hypotheses 4 and 5 are verified. Similar findings and cases in China’s PLCC practice can be obtained to support the results above. For example, Huang and Lei [50] confirm that market-based and voluntary environmental regulation policies can promote corporate green R&D investment at A-share listed companies in China. Huang et al. [57] find that bank loans and government subsidies are key factors in promoting green innovation. The larger the loan the company receives, the faster the green patent grows. In reality, as one of the first low-carbon pilot cities in China, Shenzhen has implemented strong environmental regulations in production methods, lifestyles, and project implementation and created a SMART core framework (carbon sink network, microclimate optimization, green building, low-carbon municipality, and low-carbon transportation), which has promoted green innovation rapidly. In addition, as one of the PLCC pilot cities, Chongqing focuses on enhancing urban innovation capacity through green finance. By the end of June 2022, Chongqing’s green loan and green bond balances were 478.54 billion yuan and 31.83 billion yuan, respectively, 2.7 times and 2.4 times those in early 2019. Meanwhile, the number of green patents obtained in Chongqing rose from 97 in 2010 to 729 in 2020.

By incorporating both mediating variables *reg* and *loan* into the econometric model together, we can further decompose the mediating effects. Columun (6) shows that the coefficient of policy has dropped from 0.1613 to 0.0823, which means the total intermediate effect value is 0.0790. The mediating effect values of environmental regulation and financing constraints are 0.0581 (0.0581 × 0.8292) and 0.0335 (0.0335 × 0.9391), which account for 29.88% (0.0482/0.1613 × 100%) and 19.53% (0.0315/0.1613 × 100%) of the total effect, respectively. This indicates that the environmental regulation effect plays a larger role than the financing constraint effect in promoting regional green innovation. This means that the implementation of PLCC policy brings stronger environmental regulation to enterprises. Under the dual pressure of production upgrading and emission constraints, enterprises will be more inclined to invest in green technologies, thus realizing their green innovation progress.

## 6. Conclusions and Recommendations

Based on the panel data of 285 Chinese cities from 2005 to 2020, this paper estimated the promoting effect of the PLCC policy on regional green innovation using the DID method and obtained the following four main conclusions: (1) The implementation of the PLCC policy significantly promotes green innovation, increasing the number of green patent applications in the pilot cities by about 0.1613% per year. (2) In terms of the temporal dimension, the impact of the PLCC policy on regional green innovation is characterized as an inverted “U”-shaped change in time, and the promoting effect is significant for seven years after its establishment and starts to decline in the eighth year. (3) From the spatial dimension, there is a significant spatial spillover effect of the PLCC policy on green innovation, and the indirect effect is larger than the direct effect. The spatial spillover effect ranges within 200 km of the pilot city and gradually decreases as the distance increases. (4) The PLCC policy can promote regional green innovation by enhancing environmental regulations and alleviating financing constraints. Based on the above findings, this paper puts forward corresponding recommendations.

Firstly, given that the PLCC policy may support urban green innovation and spread green technology advancement in the neighborhood, which indicates the implementation of the PLCC is currently a feasible option for China’s regional environmental policies. Therefore, policy makers can further promote the PLCC policy nationwide by refining pilot experiences and developing typical cases, contributing to the goals of “carbon peak” by 2030 and “carbon neutrality” by 2060 at the city level. In view of the weak constraints of the pilot policy, in order to fully encourage businesses to innovate in green technology and to achieve a win-win situation of low-carbon emission reduction and economic development through green technological progress, the government should effectively supervise and guide the pilot cities during the implementation of the pilot program.

Secondly, given that the policy effects of the PLCC policy are only effective for seven years, the policy effects of the PLCC should be strictly implemented based on the continuous maintenance of the sustainability of low-carbon pilot policy in relation to environmental regulation and enterprise financing to prolong the duration of the policy effects of low-carbon city pilot projects. Given that the spatial spillover effect of low-carbon city pilot policies is effective within 200 km and the spatial distribution of the existing low-carbon city pilots is still uneven, we suggest that the central government should give priority to supporting Zhengzhou, the central city of the Central Plains Economic Zone, and Heilongjiang and Changchun, the core cities of the northeast heavy industrial zone, to declare low-carbon city pilot policies. In order to foster high-quality urban development while realizing shared progress in green technology, the regional government should encourage intra-regional collaboration in green technology innovation. In particular, it should encourage peer-to-peer technology cooperation between cities with higher levels of green production.

Finally, we should value the intermediate function that environmental legislation and green financing play in the development of green technology advancement. When encouraging businesses to boost their investment in green R&D, it is essential to utilize the synergistic innovation function of various policy tools in environmental regulation. In the process of implementing pilot policies for low-carbon cities, on the one hand, policies should be designed in a targeted manner for the role subjects of different policy tools; on the other hand, the synergistic use of several policy tools should be advocated so that the effects of various policy measures on green technology innovation can be fully realized. In terms of green finance, the government should optimize and improve urban green credit financial policies to alleviate enterprise and other innovative subjects’ financing constraints. (1) The local government should establish specific funds for low-carbon development, build varied and sustainable green credit guarantee mechanisms, and innovate green low-carbon investment and financing modes. (2) Green finance should be increased to invest in low-carbon technology research and development, while financial subsidies, tax concessions, and social donations should be used to ease the financing constraints of enterprises and other innovative subjects. (3) Green credit funds ought to encourage the growth of low-carbon enterprises, especially in industries, scientific research institutions, and enterprises that tackle low-carbon core technologies, in order to accelerate the development of low-carbon core technologies and thus enhance urban green technology innovation.

## Figures and Tables

**Table 1:** Low-carbon city pilot policy process.

Pilot Starting Time	Pilot Batch	Pilot Cities
2010	First batch	Chongqing, Tianjin, Baoding, Guiyang, Hangzhou, Shenzhen, Nanchang, Xiamen
2012	Second batch	Yan’an, Suzhou, Kunming, Zunyi, Ganzhou, Jilin, Jinchang, Shanghai, Wuhan, Ningbo, Qingdao, Qinhuangdao, Guilin, Guangzhou, Wenzhou, Zhenjiang, Urumqi, Jingdezhen, Shijiazhuang, Jincheng, Nanping, Hulunbeier, Beijing, Huai’an, Chizhou, Guangyuan
2017	Third batch	Chenzhou, Liuzhou, Shenyang, Jiujiang, Ankang, Yantai, Huangshan, Jiuquan, Lanzhou, Huabei, Changsha, Hehe, Wuhai, Quzhou, Pu’er, Jiaxing, Xuancheng, Ji’an, Jinhua, Nanjing, Yuxi, Sanya, Xiangtan, Yichang, Jinan, Weifang, Yinchuan, Zhongshan, Changzhou, Zhuzhou, Sanming, Fuzhou, Dalian, Chengdu, Xining, Liu’an, Hefei

**Table 2:** Descriptive statistics of the variables.

Variables	Obs.	Max.	Min.	Mean	S.D.
*policy*	4560	1.0000	0.0000	0.13323	0.0111
*pgdp*	4560	13.0531	4.4253	9.0822	1.0824
*rd*	4560	0.0583	0.0011	0.0153	0.0012
*urb*	4560	0.9528	0.2755	0.5587	0.0911
*fdi*	4560	0.3891	0.0000	0.0351	0.0323
*gov*	4560	0.5322	0.0826	0.1454	0.9255
*hum*	4560	0.2161	0.0148	0.0755	0.0281
*tra*	4560	5.3124	2.1242	8.1244	1.3633

**Table 3:** Baseline model regression results.

Variables	(1)	(2)	(3)	(4)
*policy*	0.1843 ***	0.1724 ***	0.1775 ***	0.1613 ***
	(0.0112)	(0.0121)	(0.0075)	(0.0042)
*pgdp*			0.0685 **	0.0534 ***
			(0.0318)	(0.0137)
*rd*			0.0532 ***	0.0381 ***
			(0.0135)	(0.0114)
*urb*			0.0072 ***	0.0121 *
			(0.0021)	(0.0102)
*fdi*			−0.0871 ***	−0.0714 ***
			(0.0211)	(0.0151)
*gov*			0.5251 ***	0.4594 ***
			(0.0131)	(0.0131)
*hum*			1.2152 ***	1.1137 ***
			(0.0051)	(0.0054)
*tra*			−0.2514 **	−0.2318 *
			(0.1231)	(0.1592)
constant	−6.0101 ***	−6.2147 ***	−4.2671 ***	−5.1347 ***
	(0.8173)	(0.6647)	(0.8124)	(0.7136)
Urban fixed effects	No	Yes	No	Yes
Time fixed effects	No	Yes	No	Yes
Observations	4656	4656	4656	4656
Adjusted R^2^	0.5251	0.5612	0.5863	0.5875

Note: There are three levels of significance: The symbols ***, **, and * represent the significance at the 1%, 5%, and 10% levels, respectively. The values in parentheses are standard errors statistics value.

**Table 4:** Spatial DID model regression results.

	(1)	(2)	(3)	(4)	(5)	(6)
Variables	SAR-SDID		SEM-SDID		SDM-SDID	
	*Wd*	*We*	*Wd*	*We*	*Wd*	*We*
*policy*	0.1242 ***	0.1194 ***	0.1297 ***	0.1288 ***	0.1381 ***	0.1442 ***
	(0.0217)	(0.0121)	(0.0101)	(0.0080)	(0.0231)	(0.0223)
*W* × *policy*					0.223 ***	0.234 **
					(0.021)	(0.100)
*W* × *μ*			0.1091	0.1252 **		
			(0.0230)	(0.0501)		
*W* *×* *green*	0.0122 ***	0.0271 *			0.1121 ***	0.1011 ***
	(0.0030)	(0.0180)			(0.0100)	(0.0312)
Control variables	Yes	Yes	Yes	Yes	Yes	Yes
Urban fixed effects	Yes	Yes	Yes	Yes	Yes	Yes
Time fixed effects	Yes	Yes	Yes	Yes	Yes	Yes
Observations	4560	4560	4560	4560	4560	4560
Adjusted R^2^	0.3451	0.3853	0.3124	0.3638	0.4187	0.4464

Note: There are three levels of significance: The symbols ***, **, and * represent the significance at the 1%, 5%, and 10% levels, respectively. The values in parentheses are standard errors statistics value.

**Table 5:** Direct, indirect, and total effects of the SDM-SDID model.

		(1)	(2)	(3)	(4)
Different Effects	Variables	*Wd*		*We*	
		Coefficient	*p* Value	Coefficient	*p* Value
Direct effects	policy	0.1814 ***	0.0010	0.1761 ***	0.0010
Indirect effects	policy	0.2364 **	0.0231	0.2064 ***	0.0010
Total effects	policy	0.4178 ***	0.0041	0.3825 ***	0.0000

Note: There are three levels of significance: The symbols ***, **, and * represent the significance at the 1%, 5%, and 10% levels, respectively.

**Table 6:** Regression results of PSM-DID method.

Variables	(1)	(2)	(3)	(4)
*policy*	0.2191 ***	0.1923 ***	0.1850 ***	0.1581 ***
	(0.0231)	(0.0172)	(0.0051)	(0.0021)
constant	−8.4568 ***	−7.0164 ***	−5.4264 ***	−5.1333 ***
	(0.5473)	(0.7531)	(0.6244)	(0.4375)
Control variables	No	No	Yes	Yes
Urban fixed effects	No	Yes	No	Yes
Time fixed effects	No	Yes	No	Yes
Observations	4560	4560	4560	4560
Adjusted R^2^	0.525	0.536	0.561	0.577

Note: There are three levels of significance: The symbols ***, **, and * represent the significance at the 1%, 5%, and 10% levels, respectively. The values in parentheses are standard errors statistics value.

**Table 7:** Results of variation sample and outlier test.

Variables	(1)	(2)	(3)	(4)	(5)
*policy*	0.2132 **	0.1341 ***	0.1190 ***	0.2411 ***	0.2017 **
	(0.0922)	(0.0003)	(0.0008)	(0.0045)	(0.0841)
Control variables	Yes	Yes	Yes	Yes	Yes
Urban fixed effects	Yes	Yes	Yes	Yes	Yes
Time fixed effects	Yes	Yes	Yes	Yes	Yes
Provincial fixed effects	No	No	No	Yes	Yes
Province × time fixed effects	No	No	No	No	Yes
Observations	4560	4560	4560	4560	4560
Adjusted R^2^	0.5261	0.5851	0.6777	0.7191	0.8121

Note: There are three levels of significance: The symbols ***, **, and * represent the significance at the 1%, 5%, and 10% levels, respectively. The values in parentheses are standard errors statistics value.

**Table 8:** Results of intermediary mechanism test.

Variables	(1)	(2)	(3)	(4)	(5)	(6)
	*green*	*reg*	*green*	*loan*	*green*	*green*
*policy*	0.1613 ***	0.8292 ***	0.1112 ***	0.9391 ***	0.1255 ***	0.0823 **
	(0.0118)	(0.0243)	(0.0247)	(0.0171)	(0.0045)	(0.0412)
*reg*			0.0613 ***			0.0581 ***
			(0.0030)			(0.0111)
*loan*					0.0394 ***	0.0335 ***
					(0.0091)	(0.0100)
Control variables	Yes	Yes	Yes	Yes	Yes	Yes
Urban fixed effects	Yes	Yes	Yes	Yes	Yes	Yes
Time fixed effects	Yes	Yes	Yes	Yes	Yes	Yes
Observations	4560	4560	4560	4560	4560	4560
Adjusted R2	0.5256	0.5615	0.6747	0.5362	0.6523	0.6881

Note: There are three levels of significance: The symbols ***, **, and * represent the significance at the 1%, 5%, and 10% levels, respectively.

## Data Availability

Not applicable.
